# Comparison of One-Stage and Two-Stage Intraoperative Uterine Artery Embolization during Cesarean Delivery for Placenta Accreta: Report of Two Clinical Cases at a Tertiary Referral Medical Center

**DOI:** 10.3390/healthcare10050774

**Published:** 2022-04-22

**Authors:** Zhu-Wei Lim, Wei-Yang Lee, Yuan-Chun Huang, Wan-Ju Wu, Ming Chen

**Affiliations:** 1 Department of Obstetrics and Gynecology, Changhua Christian Hospital, Changhua 50006, Taiwan; 183434@cch.org.tw (Z.-W.L.); 180336@cch.org.tw (W.-J.W.); 2 Department of Medical Imaging, Changhua Christian Hospital, Changhua 50006, Taiwan; 155556@cch.org.tw (W.-Y.L.); 129900@cch.org.tw (Y.-C.H.); 3 Programs in Translational Medicine, National Chung Hsing University, Taichung 40227, Taiwan; 4 Department of Genomic Medicine and Center for Medical Genetics, Changhua Christian Hospital, Changhua 50046, Taiwan; 5 Department of Medical Research, Changhua Christian Hospital, Changhua 50006, Taiwan; 6 Department of Biomedical Science, Dayeh University, Changhua 51591, Taiwan; 7 Department of Obstetrics and Gynecology, College of Medicine, National Taiwan University, Taipei 10041, Taiwan; 8 Department of Medical Genetics, National Taiwan University Hospital, Taipei 10041, Taiwan; 9 Department of Post-Baccalaureate Medicine, College of Medicine, National Chung Hsing University, Taichung 40227, Taiwan

**Keywords:** morbidly adherent placenta, placenta accreta spectrum (PAS), uterine artery embolization (UAE), multidisciplinary team, hybrid OR

## Abstract

Placenta accreta spectrum (PAS) described the anchoring placental villi attached or penetrating into/through the myometrium. PAS is clinically important because of the unpredictable bleeding amount when manually removing the defective decidualization at the endometrial-myometrial interface. Therefore, a multidisciplinary strategy for cesarean delivery with PAS is crucial. Postoperative embolization after cesarean hysterectomy in a hybrid suite was studied by many scientists. In this study, we demonstrated two cases of intraoperative embolization without hysterectomy in a hybrid operating room for cesarean delivery with placenta accreta. Our results show that intraoperative uterine artery embolization with a hybrid suite is a time-preserving and safe method for cesarean delivery with PAS owing to avoiding the risk of morbidity and mortality during patient transfer.

## 1. Introduction

Morbidly adherent placenta, accreta syndromes, or placenta accreta spectrum (PAS), were the terms used to describe abnormal placental adherence and implantation [1]. PAS usually led to disastrous obstetrics outcomes due to the complications of hemorrhage, intraoperative urinary tract injury, the need for a blood transfusion, and intensive care unit admission [2]. The incidence of PAS increased remarkably from 1 in 2500 births (1980–1989), 1 in 731 births (2008–2011) and 9 in 1000 (2017–2018) because of the increasing rate of one or more repeat cesarean sections with or without placenta previa [2,3,4]. 

Management of PAS can be roughly divided into emergency delivery or planned delivery according to gestational age, labor, and bleeding severity. However, hysterectomy was one of the worst outcomes of PAS with or without emergent intervention. Therefore, scientists have demonstrated several approaches to control bleeding before the delivery of the placenta, including uterine devascularization with embolization or ligation, and balloon placement [5,6]. For those women whose placenta was invaded by an adjacent organ, leaving the placenta in situ followed by delayed hysterectomy was the most common management to control the massive amount of blood loss [7]. Other than that, conservative treatment by waiting for absorption of placenta tissue was another option for fertility preservation [1] if the individual did not complicate with infection.

Uterine devascularization with uterine artery embolization before the management of the placenta in a hybrid operating room (OR) was more feasible and safer in comparison to a conventional OR [8]. In this study, we compared the conservative management of PAS in a conventional OR (two-step procedure) versus a hybrid OR (one-step intervention) in two placenta accreta cases.

## 2. Case Report

### 2.1. Case 1

#### 2.1.1. Patient Information

The patient was a 32-year-old married woman, Gravida 1, Para 0 female with a history of deep vein thrombosis of the left leg (total occlusion of left external iliac vein, left common femoral vein and left superficial femoral vein) on Clexane therapy which was diagnosed at 8 weeks of gestation and endometrioma status post (s/p) cystectomy. She got pregnant via in vitro fertilization and frozen embryo transfer (IVF-ET). 

#### 2.1.2. Clinical Findings and Diagnostic Assessment

**Figure 1:**
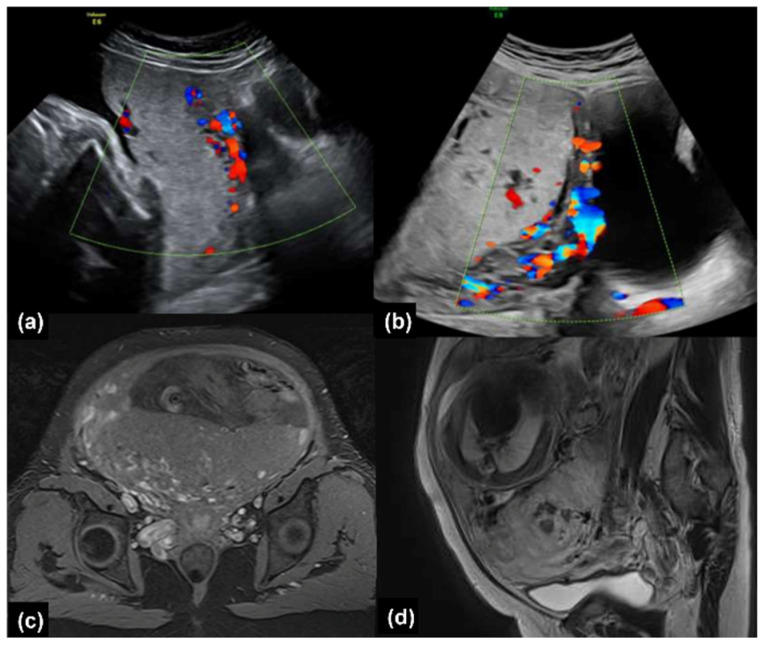
(**a**) Color Doppler ultrasound examination of abnormal placental lacunae and uterovesical hypervascularity (Case 1), and (**b**) abnormal placental lacunae, placenta bulging and uterovesical hypervascularity (Case 2). (**c**) T1-weighted transverse magnetic resonance images (MRI) demonstrating prominent vascularity around the uterus (Case 1). (**d**) T1-weighted sagittal MRI demonstrating low signal intensity area in the placenta (Case 2).

Placenta previa has been noted since the second trimester. Sonography showed abnormal placental lacunae and uterovesical hypervascularity (Figure 1a). Magnetic resonance images (MRI) showed prominent vascularity around the uterus (Figure 1b). Due to placenta previa and suspected placenta accreta, a scheduled Cesarean section was arranged at 36 weeks and 2 days. 

#### 2.1.3. Therapeutic Intervention

**Figure 2:**
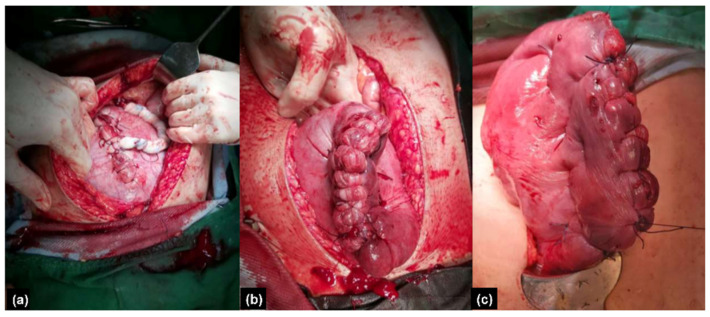
(**a**) Cord fixed at uterus (Case 1). (**b**,**c**) Repaired the uterus with interrupted sutures to approximate the uterine walls (Case 2).

**Figure 3:**
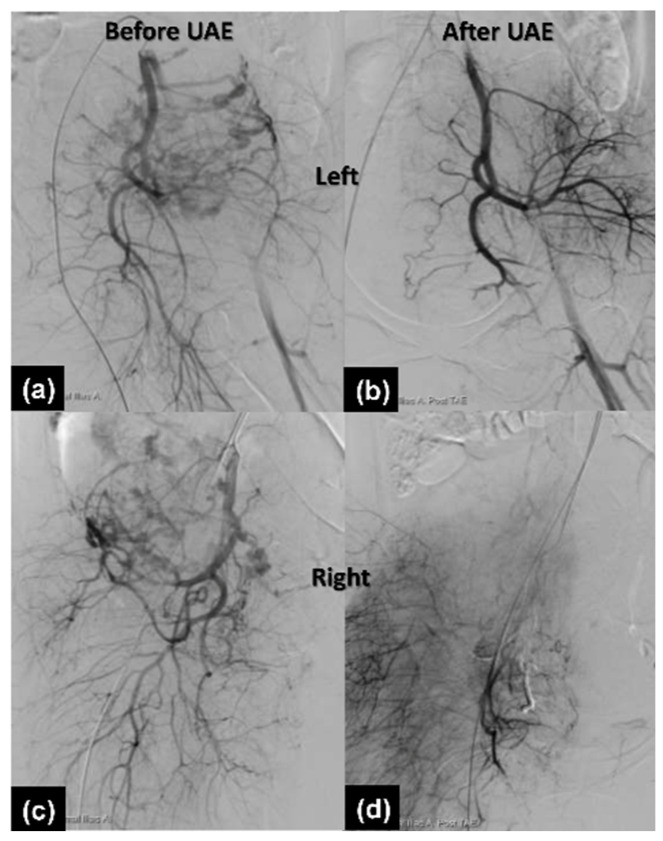
Uterine angiogram before and after the procedure (left to right). (**a**,**b**) Left. (**c**,**d**) Right.

The preoperative workup was unremarkable. A two-step procedure was performed with the classical cesarean section first. A female infant was born with a bodyweight of 3038 g and the 1 min and 5 min Apgar scores were 8 and 9, respectively. After delivery of the baby, the uterus was repaired with 1-0 Vicryl by a one-layer continuous suture method. We left the placenta in situ and fixed the umbilical cord at the uterus by a compression suture (Figure 2a) for hemostasis. Grossly engorged vessels at the lower segment of the uterus were observed. Due to maternal tachycardia and low blood pressure (70/35 mmHg), two units of red blood cells were transfused. After closing the fascia and skin, the patient was then sent to the radiology department for thrombolytic artery embolization (TAE). In the angio room, the prominent vascular flow of bilateral uterine arteries was seen (Figure 3a,c). Bilateral uterine arteries are embolized with gel foam particles mixed with cefazolin until vascular flow stasis. Post TAE angiographies revealed a significant reduction of vascular flow at bilateral uterine arteries (Figure 3b,d). She felt severe pain on the transferal. After the procedure, the patient was sent back to the operation room. Under general anesthesia, we delivered the placenta manually and did the interrupted sutures to approximate the uterine walls by the modification of the B-Lynch and Nausicaa’s compression suture [9,10]. A rubber drainage tube was inserted to prevent postpartum hemorrhage. Her vital signs were stable and the total blood loss with amniotic fluid was approximately 300 cc. 

#### 2.1.4. Follow-Up and Outcomes

After the operation, Transamin and Cefoxitin were given for hemostasis and wound infection prophylaxis. However, she felt mild dyspnea after being transferred from the post-operation room to the ward. Oxygen saturation showed 91%. We immediately gave her an oxygen mask at 6 L/min for support. Fever was noted on day 1 post-operation and post-operative atelectasis was highly suspected. Chest X rays showed mild infiltration and blunt costophrenic angles. We added acetylcysteine for mucolytic and encouraged deep breathing. O_2_ and deep breathing encouragement were provided for 1 day. Then, her fever subsided and saturation showed 98% at room air. From the third to the fifth postoperative day, C-reactive protein (CRP) improved from 10.33 mg/dL to 3.58 mg/dL. The amount of serosanguineous drainage significantly decreased day by day. The rubber tube was removed on day 4 post-operation. After the condition of the patient convalesced well, she was discharged home with her baby. 

### 2.2. Case 2

#### 2.2.1. Patient Information

The patient was a 41-years-old married woman, Gravida 2, Para 1 female with a history of myomectomy, polypectomy and hyperthyroidism under medication. Her first pregnancy ended with intra-uterine fetal death at 37 weeks of gestation. During her current pregnancy, she got pregnant by IVF-FET and placenta previa was noted in the 3rd trimester. 

#### 2.2.2. Clinical Findings and Diagnostic Assessment

Sonography showed abnormal placental lacunae, placenta bulging, and uterovesical hypervascularity (Figure 1b). MRI demonstrated an area of low signal intensity representing hemorrhage in the placenta (Figure 1d). Under high suspicion of placenta previa totalis with placenta accreta, a scheduled cesarean section was arranged at 36 weeks in a hybrid operation room. 

#### 2.2.3. Therapeutic Intervention

**Figure 4:**
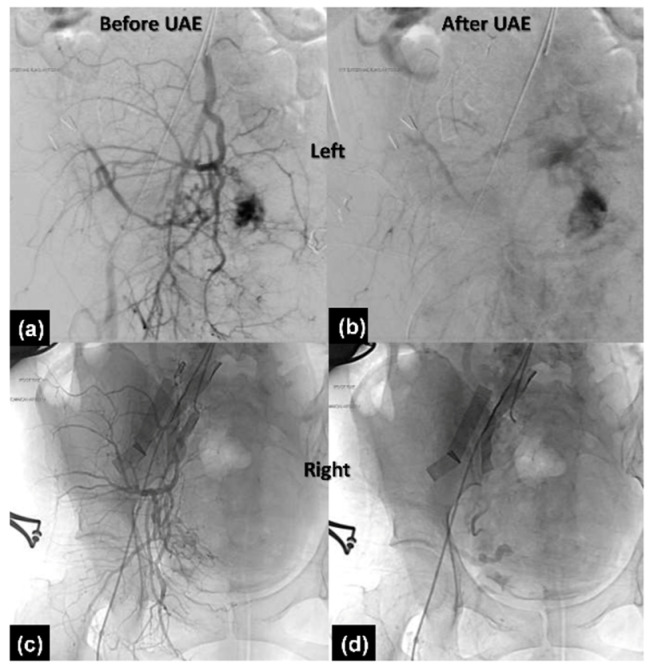
Left uterine angiography (**a**) before and (**b**) after uterine artery embolization. Right uterine angiography (**c**) before and (**d**) after uterine artery embolization.

The operation was performed smoothly via a one-step procedure. A male infant was born by classical cesarean section with a bodyweight of 2792 gm and the 1 min and 5 min Apgar scores were 8 and 9, respectively. After delivery of the baby, the uterus was repaired and the placenta remained in the uterus. TAE proceeded immediately. Angiographies of the bilateral common iliac artery and uterine artery were performed with 5 French Roberts uterine catheters via the right femoral artery approach (Figure 4a,c). Gel foam particles mixed with cefazolin were used for embolization of bilateral uterine arteries until vascular flow stasis (Figure 4b,d). Then, the placenta was manually delivered immediately after TAE. However, some placenta tissue was retained due to the minimal invasion of the myometrium. Interrupted sutures to approximate the uterine walls by methods described previously were performed smoothly [9,10] (Figure 2b,c) and a rubber tube was used for maintenance and monitoring of hemostasis. Her vital signs were stable and the total blood loss with amniotic fluid was approximately 400 cc.

#### 2.2.4. Follow-Up and Outcomes

After the operation, cefazolin and gentamycin were used as empirical antibiotics for wound infection. Laboratory data on day 3 showed Hb 8.9 g/dL, white blood cell (WBC) 12,400/μL, and CRP 13.52 mg/dL. An iron supplement was used for anemia due to acute blood loss. The antibiotic was shifted to oral form after the improvement of WBC count (6600/μL) and CRP (4.04 mg/dL) on post-operative day 5. The rubber tube was removed when little drainage amount was obtained. She was discharged in a stable condition and the out-patient appointment was scheduled within one week.

## 3. Discussion

A morbidly adherent placenta, now called the placenta accreta spectrum (PAS), is a broad term to describe the anchoring placental villi attached or penetrating into/through the myometrium [1]. The prevalence of the placenta accreta spectrum was increased nowadays due to repeated cesarean delivery [11]. Several demographic factors and clinical characteristics may contribute to PAS, including maternal age, multiparity, cigarette smoking, prior uterine incision, placenta previa and assisted reproductive technology (ART) [12,13,14,15,16]. In the classification of PAS, placenta accreta was more frequent than placenta increta and percreta, and it seemed to be less dangerous. However, until now, there were no highly specific sonography features that can be used to distinguish those items accurately.

The variant sonography features included the loss of the hypoechoic plane, abnormal placental lacunae, interruption in the bladder wall, myometrial thinning, focal exophytic mass, and bridging vessels between uterovesical and subplacental hypervascularity [17]. Recently, a “rail sign” that had a higher sensitivity (80.0%) and negative predictive value (95.1%) for PAS was introduced [16]. It was defined as two parallel neovascularization and perpendicular interconnecting bridging vessels between the bladder mucosa and placenta by color Doppler sonography [17]. Shih JC et al. have demonstrated that a positive rail sign had a significantly higher risk of placenta increta or percreta, greater perioperative approaches (preoperative vascular control, uterine artery embolization), and adverse clinical outcomes (blood transfusion, ICU admission, hysterectomy and bladder invasion) [9]. 

Early identification of the risk factors of PAS and providing extensive prenatal, peripartum and post-partum care were very crucial in the clinical practice. How to approach and remove the defective decidualized placenta at the endometrial–myometrial interface was the key factor to reducing maternal morbidity and mortality. In addition to the aforementioned risk factors, scientists have demonstrated that in vitro fertilization (IVF) frozen embryo transfer (ET) with hormone replacement therapy had a higher risk of PAS, compared to those with fresh ET and/or spontaneous conception. They suggested that the evaluation of endometrial thickness before ET was warranted [18]. The possible mechanisms were the excessive implantation time due to the thinner endometrium which affected the degree of vascular remodeling and trophoblastic invasion and the alteration of cellular pathways after in vitro culture [18].

UAE is a safe and valuable tool for the treatment of placenta accreta [19,20]. Few complications were reported in the literature. Early severe complications of treatment of placenta accreta by UAE were secondary postpartum hemorrhage and hysterectomy [20,21]. Early minor complications after UAE included postembolization fever, puncture site hematoma, infection and transient pain or numbness below the buttock area [22,23]. Late complications were less common, and included uterine necrosis, endometritis, secondary amenorrhea and hypomenorrhea [24,25,26].

Scientists have demonstrated non-conservative management of placenta accreta spectrum in a hybrid room with hysterectomy [27]. A hybrid suite contained a multi-axis robotic arm and sliding computed tomography scanner which enabled standardized image-guided surgery. The advantages of a hybrid operating room were time-saving and decreasing the risk of intraarterial catheter displacements resulting from repositioning and transferring the patient. An alternative approach to a hybrid room in local hospitals was a combination of a mobile C-arm in a conventional OR [27]. However, the image quality, infection control and space provided for anesthetic, surgical and neonatology equipment were inferior to in the hybrid room [8].

In our series, we compared two cases of placenta accreta who underwent intraoperative uterine artery embolization without hysterectomy in the radiology department versus a hybrid room (Table 1). In case 2, we provided immediately intraoperative uterine artery embolization after delivery of the baby. The total operation time and complication were lesser than case 1. In addition, pulmonary edema was developed in case 1. Sevoflurane was used in case 1 as general anesthetics. However, Desflurane (case 2) has a higher potential for respiratory distress than Sevoflurane (case 1). Therefore, fluid overload due to blood transfusion may be the cofounding factor of this complication [28]. 

## 4. Conclusions

In conclusion, intraoperative uterine artery embolization with a hybrid suite appeared to be a time-saving and safe method for conservative treatment of PAS owing to avoiding the risk of morbidity and mortality during patient transfer in our case report. Further studies are needed to assess the safety and efficacy of hybrid operating rooms.

## Figures and Tables

**Table 1:** The comparison of one-stage and two-stage intraoperative uterine artery embolization.

Characteristic	Classic Two-Step Procedure	One-Step Procedure
Age	32	41
Gravid Para	G1P0	G2P1
Prior uterine surgery	No	myomectomy, polypectomy
PAS sign: sonography	abnormal placental lacunae and uterovesical hypervascularity	abnormal placental lacunae, placenta bulging, and uterovesical hypervascularity
PAS sign: MRI	prominent vascularity around the uterus	low signal intensity representing hemorrhage in the placenta
Anesthesia	spinal then general by Sevoflurane	general by Desflurane
Time	338 min	280 min
Complications	blood transfusion; pulmonary edema	no

PAS: placenta accreta spectrum. MRI: Magnetic Resonance Imaging.

## Data Availability

Not applicable.
